# Standardized Hibiscus–Inulin Shot Lowers Lipid–Glucose Indices in Adults with Overweight and Obesity: 8-Week Randomized Trial

**DOI:** 10.3390/nu17223556

**Published:** 2025-11-14

**Authors:** Edgar J. Mendivil, Ingrid Rivera-Iñiguez, Laura P. Arellano-Gómez, Erika Martínez-López, César Hernández-Guerrero, Sonia G. Sayago-Ayerdi, José P. Tejeda-Miramontes

**Affiliations:** 1 Departamento de Salud, Universidad Iberoamericana Ciudad de México, Mexico City 01219, Mexico; cesar.hernandez@ibero.mx; 2 Department of Pediatrics, UCSD Center for Healthy Eating and Activity Research (CHEAR), University of California, San Diego, CA 92037, USA; 3 Department of Psychology, Education and Health, Nutrition and Food Sciences, ITESO Jesuit University of Guadalajara, Guadalajara 45604, Mexico; 4 Instituto de Nutrigenética y Nutrigenómica Traslacional, Departamento de Biología Molecular y Genómica, Centro Universitario de Ciencias de la Salud, Universidad de Guadalajara, Guadalajara 44340, Mexico; 5 Laboratorio Integral de Investigación en Alimentos, Tecnológico Nacional de México/Instituto Tecnológico de Tepic, Av. Tecnológico No 2595, Col. Lagos del Country, Tepic 63175, Mexico

**Keywords:** bioactive nutrients, cardiometabolic health, *Hibiscus sabdariffa*, human nutrition, metabolic syndrome, nutritional physiology, polyphenols, prebiotics

## Abstract

**Background:** Few trials have evaluated liquid chromatography–mass spectrometry (LC–MS)–standardized ready-to-drink formulations, and short-term responses of composite lipid–glucose indices under controlled intake remain unquantified. This study assessed 8-week changes in Atherogenic and triglyceride–glucose indices (AIP and TyG) after the consumption of a Hibiscus–inulin (HIB–INU) beverage and tested whether baseline risk modified these effects. **Methods:** A randomized, double-blind, placebo-controlled trial was conducted in adults aged 18–50 years with BMI ≥ 25 kg/m^2^ (*n* = 100: 50 per group) who consumed a daily 60 mL Hibiscus–inulin shot or sensory-matched placebo for 8 weeks. The Hibiscus–inulin shot was LC–MS–standardized to ensure reproducible exposure; co-primary outcomes were AIP and TyG (pre-specified as exploratory), and secondary outcomes were mean arterial pressure (MAP) and pulse pressure (PP). ANCOVA adjusted for baseline, age, and sex was used to estimate between-group differences and to test for interactions by baseline risk. **Results:** Adjusted 8-week differences versus placebo were −0.09 for AIP (95% CI −0.15 to −0.03; *p* = 0.004) and −0.14 for TyG (−0.26 to −0.03; *p* = 0.020). MAP and PP showed no significant differences between the groups (*p* > 0.05). Effects were larger in high-risk baseline strata. **Conclusions:** A standardized hibiscus–inulin shot produced short-term improvements in composite lipid–glucose indices without hemodynamic change. Because minimal clinically important differences for AIP/TyG have not been established for short durations, these findings reflect analytical responsiveness rather than clinical benefits, supporting their exploratory use in short-term nutrition trials and cardiometabolic monitoring.

## 1. Introduction

Cardiometabolic disorders, such as dyslipidemia, insulin resistance, and hypertension, are major contributors to the global prevalence of chronic diseases and premature mortality [1]. In the field of human nutrition, identifying early biomarkers responsive to dietary modifications remains challenging because of variable eating patterns and interindividual metabolic variability [2]. Conventional biomarkers (such as fasting glucose, total cholesterol, LDL-c, HDL-c, and triglycerides) often fail to capture the interconnected lipid–glucose regulatory pathways involved in these disorders, which typically involve simultaneous disruptions in energy metabolism [3]. This limitation constrains progress in developing predictive nutritional models that can inform early diet-based prevention strategies [4]. In recent years, composite indices, such as the Atherogenic Index of Plasma (AIP) and the triglyceride–glucose (TyG) index, have gained attention because they integrate triglycerides with HDL-c or fasting glucose, offering a combined perspective on lipid and glucose regulation [5]. Currently, these indices are regarded as exploratory cardiometabolic markers rather than clinically validated endpoints in nutrition intervention trials, and short-term (≤3 months) minimal clinically important differences (MCIDs) have not been defined [5,6]. Understanding their short-term behavior in response to controlled dietary interventions is key to establishing their analytical and clinical value as composite metabolic markers.

Functional foods that combine polyphenols and dietary fibers have been widely explored as nutritional interventions that target insulin signaling and lipid oxidation pathways [7,8]. Studies suggest that anthocyanins modulate oxidative balance and AMP-activated protein kinase (AMPK) activity, whereas inulin fermentation generates short-chain fatty acids (SCFAs) that improve insulin sensitivity and hepatic lipid homeostasis [1,3,5]. Because these mechanisms affect both lipid and glucose metabolism, composite indices such as AIP and TyG—which show consistent prognostic associations with incident atherosclerotic cardiovascular disease and major adverse cardiovascular events [5,6,9]—may be valuable tools for assessing whole-body metabolic coupling. Despite advances in mechanistic understanding, data on standardized dietary interventions reporting endpoints such as insulin resistance, lipid ratios, and vascular function remain limited [10,11]. Few clinical nutrition trials have used formulations with defined per-serving compositions, thereby limiting reproducibility and cross-study comparability [12]. Evaluating AIP and TyG responsiveness during short-term controlled interventions may reveal early metabolic adjustments in response to specific nutrient exposure.

*Hibiscus sabdariffa* L. is rich in polyphenols and organic acids and has been studied for its potential synergistic influence on lipid and glucose homeostasis. The beverage administered in this intervention was a standardized 60 mL Hibiscus–inulin (HIB–INU) formulation, the composition of which had been previously analyzed and standardized in an independent study by the same research group [13,14]. This prior analysis ensured consistent dosing and product reproducibility, enabling the current trial to focus on cardiometabolic and vascular outcomes, rather than chemical validation. Although synergistic effects of this combination have been proposed, differences in extraction methods and the absence of uniform, ready-to-consume formulations still compromise reproducibility across studies [14]. Employing a pre-standardized beverage ensured uniform exposure and allowed the evaluation of AIP and TyG pre-specified as exploratory co-primary endpoints, as indicators of short-term variations in lipid and glucose indices, rather than as validated surrogates of clinical benefit [6,15].

Following recent advances in formulation and analytical standardization, ready-to-drink preparations, such as the HIB–INU beverage used in this study, ensured dosing precision, chemical consistency, and reproducible nutrient exposure across participants. Adults with overweight and obesity represent a key population for investigating early dietary interventions to improve lipid profiles, insulin sensitivity, and blood pressure before pharmacological therapy becomes necessary [3,5,6,16]. However, the short-term behavior of composite indices, such as AIP and TyG, under controlled nutritional interventions remains insufficiently documented in human trials. Therefore, well-designed studies using standardized formulations are required to establish the clinical utility of these indices for nutritional monitoring and preventive health research.

This study aimed to evaluate short-term changes in the Atherogenic Index of Plasma (AIP) and the triglyceride–glucose (TyG) index in adults with overweight and obesity after consuming a previously standardized Hibiscus–inulin (HIB–INU) beverage, and to examine the variability in individual responses. The analysis included (i) the short-term variation in AIP and TyG under controlled intake, together with vascular measures such as mean arterial and pulse pressure, and (ii) the influence of baseline cardiometabolic indicators—including lipid ratios and insulin resistance—on these effects. It was hypothesized that both indices, pre-specified as exploratory co-primary endpoints, would detect early metabolic adjustments following the intervention. Using a pre-characterized, standardized formulation, this study ensured reproducibility and generated evidence on how composite indices reflect short-term metabolic adaptation, thereby supporting their use in nutritional assessment and preventive health research.

## 2. Materials and Methods

### 2.1. Study Design and Ethics

This randomized, double-blind, placebo-controlled clinical trial (ClinicalTrials.gov Identifier: NCT05774613) was conducted over 8 weeks at the Nutrition Clinic of ITESO Jesuit University of Guadalajara (Tlaquepaque, Jalisco, Mexico). The trial was part of an institutional research program investigating the metabolic and bioactive properties of a standardized *Hibiscus sabdariffa*–inulin beverage (patent MX/a/2022/010704).

The registered protocol corresponds to the acute-phase assessment of bioavailability and short-term metabolic responses of the same formulation. In contrast, the present study extends this evaluation to a chronic 8-week intervention under identical compositional and methodological conditions. The study protocol was approved by the Research Ethics Committee of ITESO (Approval ID: 0001DRC, 13 April 2021) and followed the principles of the Declaration of Helsinki [17]. The protocol pre-specified the Atherogenic Index of Plasma (AIP) and the triglyceride–glucose (TyG) index as exploratory co-primary outcomes. All participants provided written informed consent before enrollment and were informed of the study objectives, potential risks, and expected benefits.

**Figure 1:**
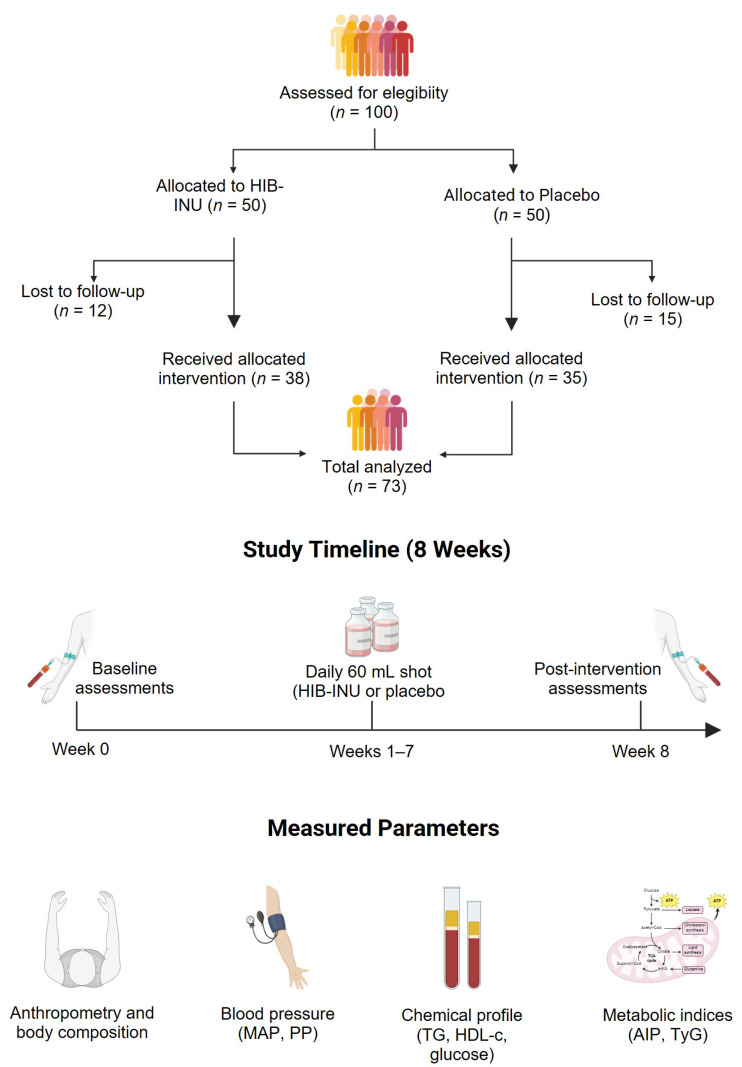
Design of the randomized, double-blind, placebo-controlled clinical trial. The diagram summarizes participant allocation, the 8-week intervention timeline, and the clinical assessments performed at baseline and post-intervention.

The overall study design, participant allocation, and timelines are shown in Figure 1. The diagram summarizes the screening and randomization process, the distribution of participants across the intervention and placebo groups, the 8-week follow-up period, and the parameters evaluated at baseline and post-intervention.

### 2.2. Participants

Men and women aged 18–50 years with a body mass index (BMI) ≥ 25 kg/m^2^ were recruited through institutional advertisements. Exclusion criteria included pregnancy or lactation, diagnosis of type 2 diabetes mellitus or other chronic non-communicable diseases, and use of medications affecting lipid or glucose metabolism. Additional exclusion criteria included adherence below 80%, insufficient or degraded biological samples, withdrawal of consent, and confirmed COVID-19 infection during the intervention [18]. Each participant was assigned a unique anonymous code to ensure data confidentiality and a consistent follow-up throughout the study.

### 2.3. Randomization and Blinding

A total of 100 participants were screened for eligibility and randomly assigned in a 1:1 ratio to the intervention (HIB–INU) or placebo groups (*n* = 50 per group). Stratified randomization was used by sex, age, and BMI to ensure comparability between groups [19]. The randomization sequence was computer-generated and managed by a researcher independent of data collection and analysis to prevent selection bias. Both beverages were distributed in identical, coded containers that were indistinguishable in terms of color, taste, and appearance. Participants, clinical staff, and data analysts remained blinded until database lock, and allocation concealment was preserved throughout the trial.

### 2.4. Intervention and Adherence

Participants in the intervention group consumed one 60 mL Hibiscus sabdariffa–inulin (HIB–INU) shot daily for 8 weeks. The placebo group received a sensory-matched beverage with a similar flavor, color, and acidity, but only trace levels of organic and phenolic compounds. Participants collected a weekly supply of seven bottles and returned the empty containers at each visit to verify compliance. Adherence was considered acceptable when the participants consumed at least 80% of the assigned doses [20]. A single trained staff member managed the beverage distribution, collection, and participant instructions to ensure uniformity across the intervention. Participants were instructed to maintain their usual dietary and physical activity habits throughout the 8-week period. Compliance with this instruction was verbally verified during weekly visits, and brief nutritional and physical activity assessments were collected at baseline and week 8 to confirm behavioral stability.

### 2.5. Beverage Preparation and Composition

The HIB–INU and placebo beverages were prepared at the Laboratorio Integral de Investigación en Alimentos (TecNM/Instituto Tecnológico de Tepic, Tepic, Mexico) under patent MX/a/2022/010704. Preparation included decoction of dried *H. sabdariffa* calyces, followed by filtration, centrifugation, and the addition of citric acid, mint, and stevia (rebaudioside A, 97% purity), as described by Sayago-Ayerdi et al. [14] and Arce-Reynoso et al. [13].

### 2.6. Sample Size Determination

The sample size was determined a priori using total cholesterol (TC) as the reference variable for power estimation, based on variance data from previous dietary interventions in adults with obesity [21]. Assuming a between-group difference of 9.41 mg/dL, standard deviation of 13.2 mg/dL, α = 0.05, and power of 80%, the required sample size was 31 participants per group. To account for attrition, 50 participants per group (*n* = 100) were recruited. After follow-up, 38 and 35 participants remained in the intervention and placebo groups, respectively. A post hoc analysis indicated that the final sample retained approximately 86% power to detect the observed difference.

### 2.7. Measurements and Outcomes

Clinical evaluations were performed at baseline (week 0) and at the end of the intervention (week 8). Participants arrived after an overnight fast of 10–12 h. Venous blood samples were obtained from the antecubital vein by qualified personnel using sterile, single-use materials. Serum and plasma were separated by centrifugation (10 min, 3500 rpm, 4 °C) and stored at −80 °C until analysis to preserve biochemical integrity [22].

Serum glucose, total cholesterol (TC), high-density lipoprotein cholesterol (HDL-c), low-density lipoprotein cholesterol (LDL-c), very-low-density lipoprotein cholesterol (VLDL-c), and triglyceride (TG) levels were determined using enzymatic colorimetric assays (Spinreact^®^, Girona, Spain). Blood pressure was measured in triplicate after a 5 min seated rest using an automated sphygmomanometer (Omron HEM-907XL^®^, Kyoto, Japan). Mean arterial pressure (*MAP*) and pulse pressure (*PP*; mmHg) were calculated using Equations (1) and (2):

(1) $$MAP=P_{d}+\frac{\left(P_{s}-P_{d}\right)}{3}$$

(2) $$PP=P_{s}-P_{d}$$

where *P_s_* and *P_d_* represent the systolic and diastolic pressures, respectively (mmHg). The primary outcomes were pre-specified changes in the Atherogenic Index of Plasma (*AIP*) and the triglyceride–glucose (*TyG*) index after 8 weeks, considered exploratory due to the lack of short-term clinical validation, and were not interpreted as a validated surrogate of clinical benefit [6,15]. Their selection was informed by prior observational and meta-analytic evidence linking these indices with incident atherosclerotic cardiovascular disease (ASCVD), CAD, and major adverse cardiovascular events [5,6,9], calculated as:

(3) $$AIP={log}_{10}\left(\frac{TG}{{HDL}_{c}}\right)$$

(4) $$TyG=ln\left(\frac{TG\times Glucose}{2}\right)$$

where *TG* and *HDL-c* were expressed in mmol/L for *AIP*, and *TG* and glucose in mg/dL for the *TyG* calculation [5,6,23]. Secondary outcomes included changes in *TC*, *LDL-c*, VLDL-c, *HDL-c*, *TG*, *MAP*, and *PP*.

Anthropometric measurements were obtained by trained staff following standardized protocols. Body composition was assessed using multifrequency bioelectrical impedance (InBody 770^®^, Seoul, Republic of Korea), and waist, abdominal, and hip circumferences were measured with a metallic, inextensible tape (Lufkin^®^, Missouri City, TX, USA) according to International Society for the Advancement of Kinanthropometry (ISAK) guidelines. Each measurement was performed twice to minimize technical errors and ensure reproducibility.

### 2.8. Statistical Analysis

Group comparisons of unadjusted means were assessed using Welch’s *t*-test, which accounts for potential variance heterogeneity between groups. The primary inferential analysis employed analysis of covariance (ANCOVA) with the treatment group as a fixed factor and baseline value, age, and sex as covariates to control for interindividual variability. The co-primary outcomes (ΔAIP and ΔTyG) were modeled using the ANCOVA specification. Adjusted effects were expressed as *β*-coefficients with 95% confidence intervals and associated *p*-values. Participants meeting the predefined thresholds of AIP < 0.24 and PP < 50 mmHg at week 8 were classified as responders [2,24]. These indicators were used for descriptive responder summaries and were not used in the PCA or to derive PCA-based profiles.

Principal component analysis (PCA) was applied to the z-score-standardized changes (ΔAIP, ΔTyG, ΔMAP, and ΔPP) to describe the overall structure of the cardiometabolic response. Components with eigenvalues greater than one were retained following Kaiser’s criterion and verified by parallel analysis [25,26]. Component loadings and cumulative explained variance were reported, and projections onto PC1 and PC2 were used to depict participants’ distribution according to their metabolic–hemodynamic response profiles.

PCA-based profiles were identified post hoc using a quadrant-based rule on the signs of individual PC1 and PC2 scores to visualize multivariate response patterns. Robustness procedures include random subsampling and re-estimation using an alternative scaling specification. For visualization, a PC1–PC2 biplot was produced with group centroids (mean PC1–PC2), 95% confidence ellipses, and variable loading vectors.

A stratified analysis was conducted to determine whether baseline cardiometabolic status influenced the magnitude of treatment effects. Participants were categorized into low, medium, and high baseline tertiles for AIP, TyG, and MAP, and all comparisons were adjusted for age and sex to examine potential treatment-by-baseline interactions.

Missing data below 5% were imputed under the missing-at-random assumption. All statistical analyses, figures, and data processing were performed using OriginPro 2025b (v10.2.5.212; OriginLab Corporation, Northampton, MA, USA) to ensure internal consistency between numerical and graphical outputs.

### 2.9. Quality Control

All study personnel received standardized training to maintain uniform fasting instructions, blood collection, and data recording. Participant identity was protected using coded identifiers, and all records were securely stored on institutional servers. Participation was voluntary, and participants could withdraw at any time without consequence. Compliance with institutional and national ethical standards was reviewed periodically to ensure adherence to good clinical practice.

## 3. Results and Discussion

### 3.1. Baseline Demographic and Clinical Characteristics

This study evaluated 73 participants (placebo, *n* = 35; HIB–INU, *n* = 38). Table 1 summarizes the anthropometric, hemodynamic, and biochemical characteristics of the study population at baseline. No statistically significant differences were detected between groups in the variables analyzed, confirming adequate randomization and baseline comparability.

### 3.2. Chemical Composition and Bioactive Properties

Table 2 presents the chemical composition and bioactive profile of the HIB–INU formulation and the placebo (per serving). The HIB–INU formulation contained 937.37 mg of bioactive compounds—primarily organic acids (90.1%) and phenolics (9.9%)—at higher levels than the placebo. Organic acids predominated (844.39 mg); Hibiscus acid was the most abundant constituent (676.90 mg). Phenolic acids totaled 44.85 mg (methyl chlorogenate II predominant); anthocyanins totaled 36.25 mg (delphinidin-3-sambubioside predominant); and flavonoids totaled 11.86 mg. The remaining solids consisted of 17.16 g of non-phenolic compounds, primarily fructans (15.38 g). The placebo contained 1.22 mg phenolics and 0.12 g non-phenolics (per serving). Relative standard deviations (RSDs) were <10% across the analytes, supporting the analytical precision.

Previous studies have identified Hibiscus acid and delphinidin-3-sambubioside as the most abundant compounds in *H. sabdariffa* extracts [11,27,28], consistent with the composition observed in the HIB–INU formulation. In Hibiscus-based matrices, these compounds have been reported to contribute to antioxidant capacity and chemical stability, with potential implications for product stability and compositional integrity; however, these effects were not assessed in this study [14]. Beyond composition, fructans may stabilize the formulation (slightly increased viscosity and reduced exposure of phenolics to O_2_), thereby minimizing degradation during storage [29]. Current evidence suggests that stability may depend on concentration and on the balance among compounds within the matrix; this hypothesis should be verified in longitudinal storage studies and in-trial sampling [30].

Although this study provided a detailed chemical characterization of a Hibiscus-based beverage, and may serve as a reference for future formulations [31], its scope was limited to initial composition and analytical precision, and did not include longitudinal stability, which constitutes a study limitation. Understanding and controlling compositional variation during storage and throughout the intervention period are essential for reproducibility and interpretation, as changes in structure or concentration can alter bioavailability and downstream biological responses. Future studies should evaluate chemical changes under different storage conditions and during interventions, as well as explore technological approaches, such as encapsulation or alternative protective systems, to preserve stability and composition in clinical and research settings.

### 3.3. Cardiometabolic Effects of the Intervention

As detailed in Section 3.2, the chemical composition was consistent with the selection of AIP and TyG as exploratory co-primary metabolic endpoints and MAP and PP as hemodynamic endpoints. Table 3 presents baseline and week-8 values and mean within-group changes; between-group comparisons were assessed using Welch’s *t*-test. After 8 weeks, the HIB–INU group showed a mean decrease of −0.083 in AIP and −0.144 in TyG, while the placebo group remained nearly unchanged (−0.002 and −0.016, respectively). The between-group differences were significant for AIP (*p* = 0.007) and TyG (*p* = 0.049), indices linked to incident ASCVD (TyG, Hazard Ratio (HR) = 1.61) and major adverse cardiovascular events (AIP, Relative Risk (RR) = 1.63) [5,6]. Mean arterial pressure decreased slightly in the HIB–INU group (−2.9%; *p* = 0.092), and pulse pressure varied by less than 1% (*p* = 0.957), indicating that hemodynamic parameters remained stable while metabolic indices improved during the 8-week intervention.

The HIB–INU pattern was consistent with prior findings from studies of *H. sabdariffa* and anthocyanin-based formulations, which have reported reductions in TG, LDL-c, and fasting glucose. Since AIP integrates TG and HDL-c and TyG integrates TG and fasting glucose, these lipid–glucose changes are directly relevant to the composite indices. Pan et al. [7] reported decreases in TG (−0.11 mmol/L), LDL-c (−0.18 mmol/L), and fasting glucose (−0.29 mmol/L) after 4–8 weeks of supplementation. Wan et al. [32] documented similar lipid responses in 284 trials, without changes in blood pressure. Ellis et al. [10] observed LDL-c reductions (−6.8 mg/dL; *p* = 0.05) and systolic blood pressure (−7.1 mmHg; *p* = 0.02), indicating concurrent but independent lipid and hemodynamic benefits of Hibiscus supplementation. Similar short-term changes were also observed in Hibiscus–verbena trials, with LDL-c declining by 4–5% and HDL-c increasing by 5% [16,33]. The magnitude and duration of the metabolic effects in this study appear to be consistent with previous reports and agree with the lack of significant MAP and PP changes during the 8 weeks [34].

The composition of HIB–INU could partially account for the metabolic effects observed in this study. The predominance of Hibiscus acid may help to maintain an acidic environment, thereby enhancing anthocyanin solubility and intestinal absorption. It could also modulate interactions with α-glucosidase and the sodium-glucose cotransporter 1 (SGLT1) in the upper small intestine, potentially contributing to a slower appearance of glucose in circulation, consistent with the observed decline in TyG [11,27]. In the liver, anthocyanins and phenolic acids have been proposed to influence AMP-activated protein kinase (AMPK) and to downregulate lipogenic enzymes, such as acetyl-CoA carboxylase (ACC) and fatty acid synthase (FAS), thereby reducing triglyceride synthesis and helping explain the lower AIP [35,36]. Inulin fermentation yields acetate and propionate, which stimulate the G-protein-coupled receptor 41 and 43 (GPR41/43); these pathways are associated with improved insulin sensitivity and lipid oxidation [37,38], consistent with the lipid–glucose pattern observed.

The observed reductions in AIP and TyG reflect early metabolic changes that align with established physiological patterns of lipid–glucose regulation, without implying a direct mechanistic link between the intervention and outcome. The combined presence of Hibiscus acid, anthocyanins, and fructans may account for these metabolic effects through complementary physiological actions, although the lack of microbiome data, circulating metabolite data, and hepatic biomarker data precludes experimental verification. Consequently, the proposed mechanisms should be regarded as exploratory hypotheses grounded in known physiological principles, rather than verified causal relationships. Given this limitation, cautious interpretation is warranted, and the following section explores whether these responses vary across baseline metabolic profiles.

### 3.4. Metabolic Response and Responder Profile

**Figure 2:**
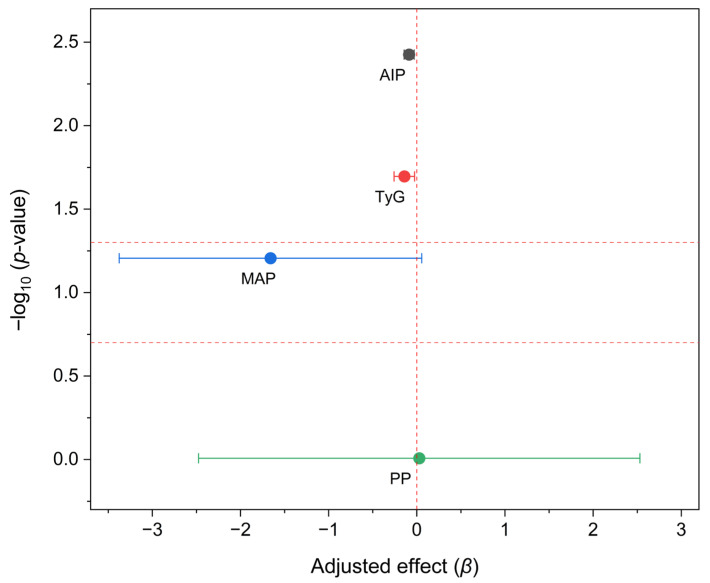
Adjusted effects of HIB–INU on cardiometabolic indices (AIP, TyG, MAP, PP). Negative *β* values indicate improvement relative to placebo. Analyses were performed by ANCOVA adjusted for baseline value, age, and sex.

Adjusted analyses indicated that the HIB–INU intervention produced short-term effects on integrated cardiometabolic markers after 8 weeks of controlled exposure. As summarized in Table 4, models adjusted for baseline, age, and sex showed reductions of −0.14 in TyG and −0.09 in AIP, both of which were statistically significant (*p* < 0.05) compared with placebo. Mean arterial and pulse pressures showed minimal variation (*p* > 0.05), suggesting that the response was primarily metabolic rather than hemodynamic. The proportion of participants with AIP < 0.24 reached nearly 74% in the HIB–INU group, compared with 63% in the placebo group, with an absolute difference of 10.8%, reflecting a modest but consistent shift toward a less Atherogenic profile. These short-term variations represent analytical rather than clinical changes, as no validated thresholds link AIP or TyG shifts of this magnitude to measurable reductions in risk. Population analyses also describe a J-shaped association between AIP and all-cause mortality (inflection ≈ 0.0905), reinforcing a cautious clinical interpretation [15]. The pattern and direction of these adjusted effects, as illustrated in Figure 2, provide a framework for interpreting TyG- and AIP-related responses.

Previous studies have consistently shown that higher TyG index values are associated with adverse cardiometabolic outcomes, including mortality and arterial stiffness [39,40]. In this study, the adjusted reduction in TyG (−0.14 ± 0.06; *p* = 0.020) was consistent with an initial improvement in lipid–glucose coupling under controlled conditions, suggesting a subtle metabolic influence of the HIB–INU beverage. Extensive cohort analyses have reported that each unit increase in TyG is linked to higher mortality risk (odds ratio (OR) = 1.68; *p* < 0.001) and greater odds of arterial stiffness (OR = 1.94; 95% CI 1.10–3.44), with mean TyG values of 9.10 ± 0.59 in affected individuals and 8.77 ± 0.57 in those without stiffness [39,40]. A meta-analysis of 17 cohorts found that each unit increase in TyG was associated with a higher risk of atherosclerotic cardiovascular disease (HR = 1.28; 95% CI 1.13–1.45) and coronary artery disease (HR = 1.39; 1.15–1.68) [5], supporting its relevance as a cardiometabolic biomarker. The observed reduction may reflect an early metabolic adjustment toward a lower-risk phenotype, without implying clinical improvement. The TyG index integrates hepatic lipogenesis and insulin resistance, and its decline could reflect subtle changes in substrate utilization preceding detectable vascular responses, which is consistent with stable mean arterial and pulse pressures.

Additionally, the adjusted reduction in AIP (−0.09 ± 0.03; *p* = 0.004) indicated parallel modulation of lipid–glucose homeostasis, consistent with the decrease observed in TyG. Higher AIP has been associated with major adverse cardiovascular events in CAD cohorts (RR = 1.63), supporting its role as a complementary risk marker rather than a stand-alone endpoint [6]. This pattern aligns with the compositional features of the HIB–INU formulation, which combines anthocyanin-rich Hibiscus and the prebiotic inulin, and can influence triglyceride and HDL balance under controlled exposure. Evidence shows that AIP increases with worsening metabolic profiles [41], with reported mean values of 0.32 ± 0.21 in metabolically unhealthy obesity (MUO), 0.24 ± 0.17 in metabolically unhealthy obese (MHO), and 0.18 ± 0.16 in metabolically unhealthy non-obese individuals [41]. Because AIP is sensitive to dietary composition and overall energy balance [4], the concurrent decreases in AIP and TyG observed in this study likely reflect early lipid remodeling toward a less Atherogenic profile during the short-term nutritional intervention, highlighting a coordinated metabolic response rather than isolated effects [42].

In this study, the overall pattern of adjusted responses supported a coherent metabolic effect of HIB–INU, complementing the reductions observed in TyG and AIP. These results indicate that lipid–glucose regulation can be influenced by controlled nutritional exposure without measurable effects on hemodynamic parameters. Although limited to 8 weeks, these data establish a clear analytical basis for examining how the response magnitude and direction vary among participants, highlighting the need for long-term follow-up with mechanistic biomarkers. This analytical focus enables a detailed exploration of the response distribution across metabolic dimensions, leading to subsequent analysis of physio-metabolic variability. While exploratory, these outcomes provide a quantitative basis for the future validation of AIP and TyG as responsive biomarkers in controlled nutrition trials.

### 3.5. Physio-Metabolic Profile and Response Variability

**Figure 3:**
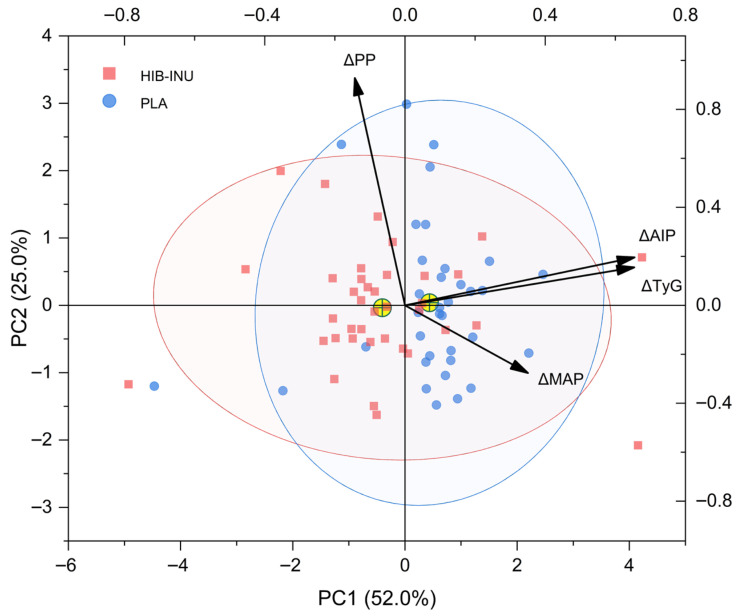
Principal component analysis of cardiometabolic response changes. Biplot of ΔAIP, ΔTyG, ΔMAP, and ΔPP for the HIB–INU (red) and placebo (blue) groups. Points are individual PC1–PC2 scores; centroids (yellow ⊕) and 95% confidence ellipses show group clustering. Arrows represent variable loadings. All variables were z-standardized before PCA; PC1 and PC2 explain 52% and 25% of the total variance, respectively.

Principal component analysis (PCA) was used to identify variables that showed similar response patterns among participants in the HIB–INU intervention. As shown in Table 5, Component 1 (PC1) displayed the highest positive loadings for ΔAIP (0.6548) and ΔTyG (0.6542), forming a metabolic component associated with cardiometabolic risk and insulin resistance. Component 2 (PC2) was mainly defined by ΔPP (0.9281), with a smaller inverse contribution from ΔMAP (–0.2761), representing a vascular component related to blood pressure control. These two components explained 77.0% of the total variance (PC1 = 52.0%; PC2 = 25.0%), summarizing most of the physio-metabolic variation observed. Scores were then stratified post hoc by a rule-based quadrant criterion on the signs of PC1 and PC2 to depict multivariate response patterns. The PC1–PC2 score plot (Figure 3) shows the distribution of individual values; participants receiving HIB–INU tended to occupy regions associated with lower AIP, TyG, MAP, and PP (see Section 3.3).

Previous studies on short-term dietary interventions have reported links between glucose–lipid regulation and vascular function [25,43]. Tiezzi et al. [43] found that 8 weeks of dietary treatment reduced fasting glucose (~8 mg/dL) and triglycerides (~20 mg/dL). The intervention also improved endothelial function (FMD +3.2%; *p* < 0.05), although blood pressure did not change significantly (*p* = 0.42). These results suggest that metabolic improvements may enhance endothelial function even in the absence of measurable hemodynamic effects. A similar pattern was observed in this study: ΔMAP and ΔPP (PC2) remained stable, whereas ΔAIP and ΔTyG (PC1) decreased, reflecting an early physio-metabolic response to HIB–INU supplementation. PCA facilitated the visualization of these coordinated effects, supporting previous evidence [25] that multivariate analysis can capture diet-related physiological changes.

Applying this rule-based quadrant classification yielded four descriptive response categories (see Table 6). Complete responders (26%) showed concurrent metabolic and vascular improvements (PC1 < 0, PC2 < 0), whereas partial responders (19%) improved mainly in ΔAIP and ΔTyG. Vascular-dominant participants (23%) showed specific changes in ΔMAP and ΔPP, while non-responders (31%) showed slight variation across parameters. This pattern aligns with the findings reported by Mittendorfer et al. [44], who studied women with about 20% weight loss and found that metabolic recovery differed—some participants improved hepatic insulin sensitivity, others peripheral insulin sensitivity, and some showed little benefit despite similar weight loss. They also indicated that baseline metabolic sensitivity influenced individual responses to dietary bioactives and emphasized the importance of considering this relationship in future investigations. Sensitivity analyses—random subsampling and re-estimation under alternative scaling—produced a similar component structure and loading pattern, supporting the robustness of the PCA solution.

The PCA results in this study indicated that metabolic and vascular responses could vary within short-term nutritional interventions. Instead of showing uniform effects, the analysis revealed a structured pattern of variability, which may reflect different physiological adjustments among participants receiving HIB–INU. This multivariate approach enhances the interpretation of integrated markers by identifying combinations that respond jointly to controlled exposure. Although the 8-week duration and the absence of circulating biomarkers limit mechanistic interpretation, these observations provide valuable insights into early metabolic adaptation and may guide future studies that consider individual metabolic sensitivity and baseline physiological status in dietary interventions.

### 3.6. Baseline Risk and Metabolic Response

**Figure 4:**
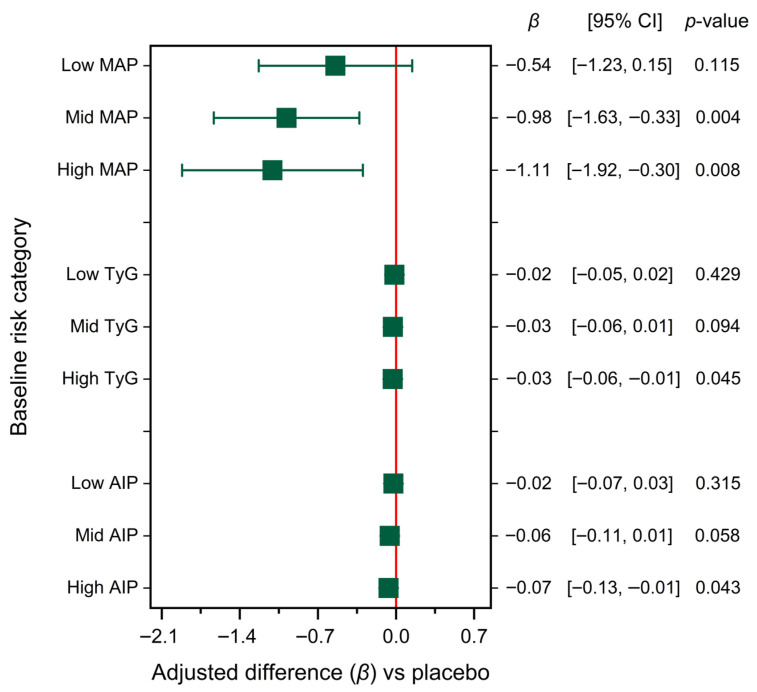
Risk-stratified effects of HIB–INU on cardiometabolic indices. Adjusted *β*-coefficients (95% CI) for 8-week changes in MAP, TyG, and AIP between HIB–INU and placebo groups (ANCOVA adjusted for baseline, age, and sex; negative *β* indicates improvement).

Participants were stratified by baseline risk level for each marker (MAP, TyG, and AIP), and analyses were adjusted for age and sex (Figure 4). This approach revealed distinct response patterns among risk categories. HIB–INU supplementation was associated with greater improvements in metabolic markers in the high-risk stratum (ΔTyG −0.03; ΔAIP −0.07), moderate changes in the intermediate-risk stratum (ΔMAP −0.98 mmHg), and negligible changes in the low-risk stratum (no change in MAP; *p* = 0.115). This gradient suggests a risk-dependent response: individuals with a higher baseline burden showed greater lipid–glucose responsiveness, and higher baseline AIP/TyG may inform monitoring intensity in nutrition-focused follow-up [5,6]. The changes in MAP were modest across all strata, with the most significant reduction observed in the high-risk group (ΔMAP −1.11 mmHg).

Comparable trends have been reported in previous interventions targeting cardiometabolic risk [45,46]. In type 2 diabetes (T2D), anthocyanin–prebiotic combinations have been associated with reductions in fasting glucose and LDL-c levels over 60 days, in alignment with the direction of effects observed in the present study [45]. In people with obesity, inulin supplementation resulted in modest improvements in blood pressure and lipid marker levels [46]. Although differences in endpoints and population characteristics limit direct comparisons, these findings provide a helpful context for interpreting the stratified responses. Evidence from clinical trials and meta-analyses supports this interpretation [12]. Pre-, pro, and symbiotic supplementation has been shown to improve fasting glucose, TG, and HDL-c levels, with limited effects on LDL-c—particularly in dysmetabolic cohorts [12]. Although these outcomes have been reviewed in detail in earlier sections, they underscore the relevance of dietary bioactive compounds for modulating metabolic parameters across risk phenotypes [8,47].

As noted previously, mechanistic biomarkers were not assessed in this study; however, based on existing evidence, microbial fermentation and SCFA production may influence insulin sensitivity and lipid metabolism, potentially contributing to the greater metabolic benefits observed in people with increased susceptibility to metabolic disorders. The short intervention period, modest sample size, and absence of longitudinal molecular endpoints limit the generalizability of these findings. In addition, since participants were adults aged 18–50 years without diagnosed metabolic disease, the results should be interpreted within this context, as responsiveness may differ in older or metabolically compromised populations, consistent with population-level J-shaped mortality patterns for AIP [15] and with glucose-status–dependent differences reported in angiographic cohorts [48]. Future studies should incorporate mechanistic metabolomics, microbiome, and vascular biomarkers into stratified models and long-term follow-up to test baseline-dependent effects and improve clinical translation.

## 4. Conclusions

This 8-week randomized, double-blind, placebo-controlled trial showed that the standardized HIB–INU beverage reduced AIP (−0.09; *p* = 0.004) and TyG (−0.14; *p* = 0.020) without significant changes in MAP or PP, indicating a specific metabolic effect. The reductions in these indices suggest early lipid–glucose adjustments that align with the beverage’s polyphenol–fructan composition. PCA revealed a dominant metabolic axis driven by AIP and TyG, while stratified analyses showed greater improvements in participants with higher baseline risk, indicating that the initial cardiometabolic status influenced the magnitude of response. Overall, these findings support AIP and TyG as analytically responsive short-term markers of diet-induced metabolic modulation. However, the short duration, modest sample size, and lack of molecular or vascular endpoints limit both mechanistic and clinical interpretation. Future trials with longer follow-up periods, including metabolomic, microbiome, and vascular analyses, are warranted to confirm these baseline-dependent effects and to define clinically meaningful thresholds for short-term changes in composite lipid–glucose indices.

## 5. Patents

The beverage formulation described in this study is protected under the Mexican patent “Bebida de Jamaica con alta concentración de antioxidantes y fibra dietética y proceso de obtención de la misma” (MX/a/2022/010704).

## Figures and Tables

**Table 1:** Baseline demographic and clinical characteristics of participants by treatment group.

Parameter	Placebo (*n* = 35)	HIB–INU (*n* = 38)	*p*-Value
Age (years)	39.37 ± 10.84 (40.0)	37.89 ± 10.76 (36.5)	0.562
Weight (kg)	71.17 ± 13.23 (74.7)	74.17 ± 15.91 (77.2)	0.383
Height (cm)	170.86 ± 11.22 (172.0)	168.21 ± 10.71 (168.0)	0.307
Body Mass Index (kg/m^2^)	25.43 ± 2.61 (24.6)	26.20 ± 3.39 (24.7)	0.136
Waist Circumference (cm)	82.47 ± 10.98 (79.4)	86.79 ± 13.28 (88.7)	0.133
Hip Circumference (cm)	100.11 ± 7.25 (101.0)	100.06 ± 9.32 (98.8)	0.977
Systolic Blood Pressure (mmHg)	111.49 ± 11.20 (109.0)	114.82 ± 13.16 (114.0)	0.247
Diastolic Blood Pressure (mmHg)	70.03 ± 9.17 (69.0)	72.97 ± 9.35 (73.0)	0.179
Glucose (mg/dL)	88.03 ± 13.90 (88.0)	90.18 ± 15.38 (88.5)	0.531
Triglycerides (mg/dL)	111.77 ± 46.74 (97.0)	102.84 ± 39.34 (93.5)	0.382
HDL-c (mg/dL)	63.46 ± 7.80 (64.0)	60.82 ± 7.93 (62.0)	0.156
Total Cholesterol (mg/dL)	187.60 ± 16.48 (189.0)	187.97 ± 18.73 (188.5)	0.928
LDL-c (mg/dL)	101.79 ± 13.92 (103.2)	106.59 ± 18.35 (107.5)	0.210

Data are presented as mean ± SD (median in parentheses). Between-group comparisons were performed using Welch’s *t*-test (two-tailed).

**Table 2:** Chemical composition and bioactive profile of HIB–INU shot and placebo (60 mL).

Component	Placebo	HIB–INU
Total bioactive compounds (mg/60 mL)	1.22 ± 0.06	937.37 ± 96.31
Organic acids (mg/60 mL)	0.86 ± 0.03	844.39 ± 86.32
Hibiscus acid	0.80 ± 0.01	676.90 ± 67.10
Hydroxycitric acid	0.06 ± 0.02	91.35 ± 9.01
Hibiscus acid dimethylester	N.D.	62.16 ± 6.18
Phenolic acids (mg/60 mL)	0.34 ± 0.03	44.85 ± 20.23
Methylchlorogenate I	N.D.	14.61 ± 1.25
Methylchlorogenate II	N.D.	23.13 ± 2.14
Flavonoids (mg/60 mL)	N.D.	11.86 ± 1.21
Quercetin-galloyl-hexoside	N.D.	4.22 ± 0.00
Myricetin-3-O-sambubioside	N.D.	3.24 ± 0.31
Anthocyanins (mg/60 mL)	N.D.	36.25 ± 3.18
Delphinidin-3-sambubioside	N.D.	23.93 ± 2.35
Cyanidin-3-glucoside	N.D.	8.43 ± 0.86
Delphinidin	N.D.	3.89 ± 0.41
Total non-phenolic solids (g/60 mL db)	0.12 ± 0.01	17.16 ± 0.27
Fructans	N.D.	15.38 ± 0.45
Fructose	0.06 ± 0.00	1.04 ± 0.07
Glucose	0.03 ± 0.01	0.53 ± 0.03
Sucrose	0.01 ± 0.00	0.03 ± 0.00
Ash	0.02 ± 0.01	0.18 ± 0.02

Values are expressed as the mean ± SD (*n* = 3). N.D.—not detected; db—dry basis.

**Table 3:** Baseline and 8-week cardiometabolic markers and between-group differences.

Variable	Group	Baseline	Week 8	Δ Mean	*p*-Value
AIP	Placebo	0.21 ± 0.20	0.21 ± 0.21	−0.002	0.007
	HIB–INU	0.20 ± 0.19	0.12 ± 0.20	−0.083	
TyG	Placebo	8.41 ± 0.44	8.39 ± 0.42	−0.016	0.049
	HIB–INU	8.36 ± 0.41	8.21 ± 0.37	−0.144	
MAP	Placebo	83.85 ± 8.53	82.81 ± 9.46	−1.038	0.092
	HIB–INU	86.92 ± 9.51	84.46 ± 9.89	−2.465	
PP	Placebo	41.46 ± 10.62	41.26 ± 11.36	−0.200	0.957
	HIB–INU	41.84 ± 10.71	41.71 ± 10.95	−0.132	

Data are mean ± SD. Δ represents the change within each group (week 8—baseline). *p*-values denote between-group comparisons (placebo vs. HIB–INU; Welch’s *t*-test). AIP—Atherogenic index of plasma; TyG—triglyceride–glucose index; MAP—mean arterial pressure (mmHg); PP—pulse pressure (mmHg).

**Table 4:** Adjusted differences and responder proportions for cardiometabolic markers.

Variable	Adjusted Effect (*β* [95% CI])	*p*-Value	Responders HIB–INU (%)	Responders Placebo (%)	ARD (95% CI)
ΔAIP	−0.09 [−0.15, −0.03]	0.004	73.7	62.9	10.8 [−10.4, 32.1]
ΔTyG	−0.14 [−0.26, −0.03]	0.020	N.A.	N.A.	N.A.
ΔMAP	−1.66 [−3.38, 0.06]	0.062	N.A.	N.A.	N.A.
ΔPP	0.03 [−2.48, 2.53]	0.983	73.7	71.4	2.3 [−18.2, 22.8]

*β* represents the adjusted between-group difference (placebo vs HIB–INU) estimated by ANCOVA controlling for baseline, age, and sex; robust standard errors were used to account for potential variance heterogeneity. Responders were defined as AIP < 0.24 and PP < 50 mmHg; ARD—absolute risk difference; CI—confidence interval; N.A.—not applicable.

**Table 5:** PCA loadings.

Variables	PC1	PC2
ΔAIP	0.6548	0.1958
ΔTyG	0.6542	0.1550
ΔMAP	0.3505	−0.2761
ΔPP	−0.1431	0.9281

PCA—principal component analysis; PC1—principal component 1; PC2—principal component 2. Δ indicates within-group changes (week 8—baseline).

**Table 6:** Classification of individual response patterns according to PCA scores.

Response Category	Criteria	*n*	% of Total (*n* = 73)
Complete responders	PC1 < 0, PC2 < 0	19	26.0
Partial metabolic responders	PC1 < 0, PC2 > 0	14	19.2
Vascularly mixed profiles	PC1 > 0, PC2 < 0	17	23.3
Non-responders	PC1 > 0, PC2 > 0	23	31.5
Total		73	100

PC1—principal component 1; PC2—principal component 2; *n*—number of participants.

## Data Availability

The data generated in this work are available upon request from the corresponding author. The data are not publicly available due to privacy restrictions.
